# Supplementary figures and images for: Defining the cellular and molecular features of nerve-invaded cancer cells using a newly characterized experimental model
Source: Cell Death Discov. 2025 Jul 8;11:314. doi: 10.1038/s41420-025-02616-4 (PMC12238365; doi:10.1038/s41420-025-02616-4)

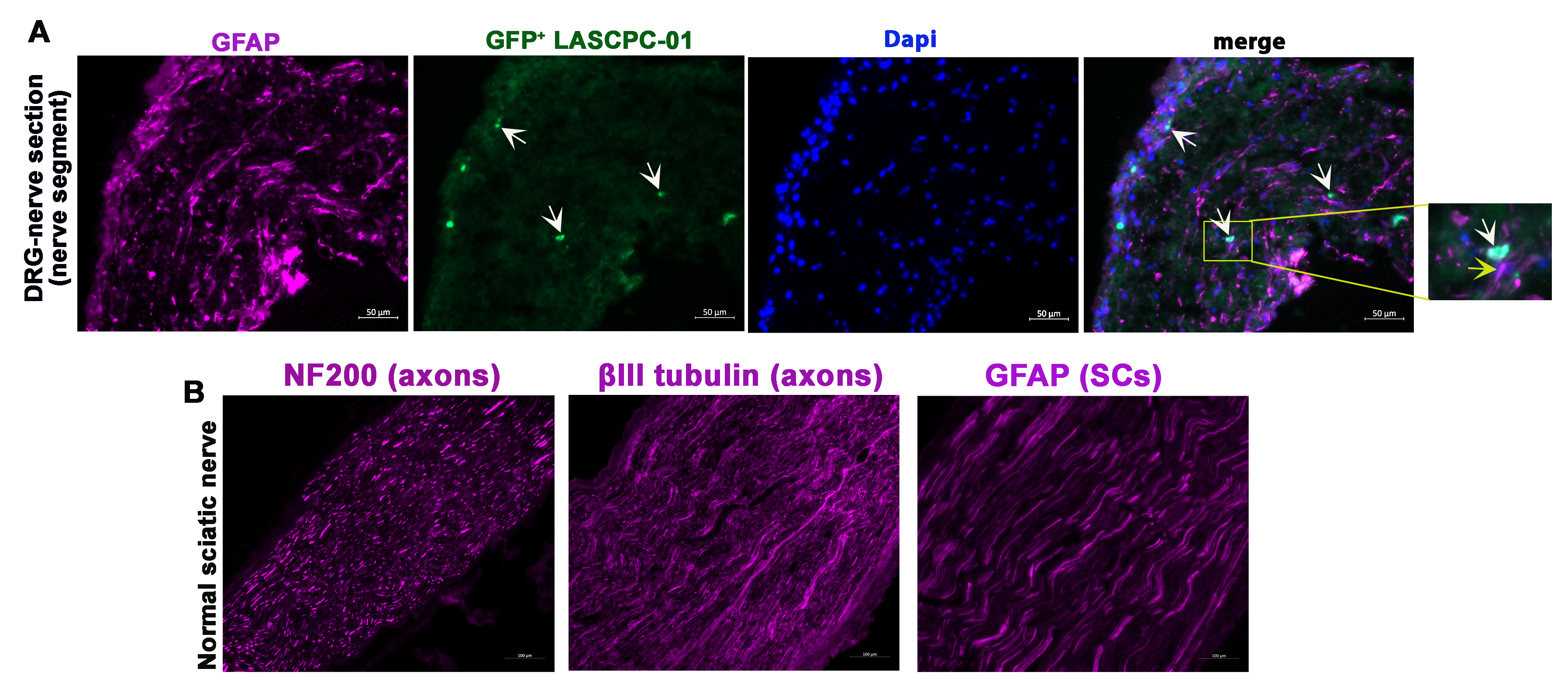

Supplement: Supplementary file 2 — Figure S1 [file 41420_2025_2616_MOESM2_ESM.tif]

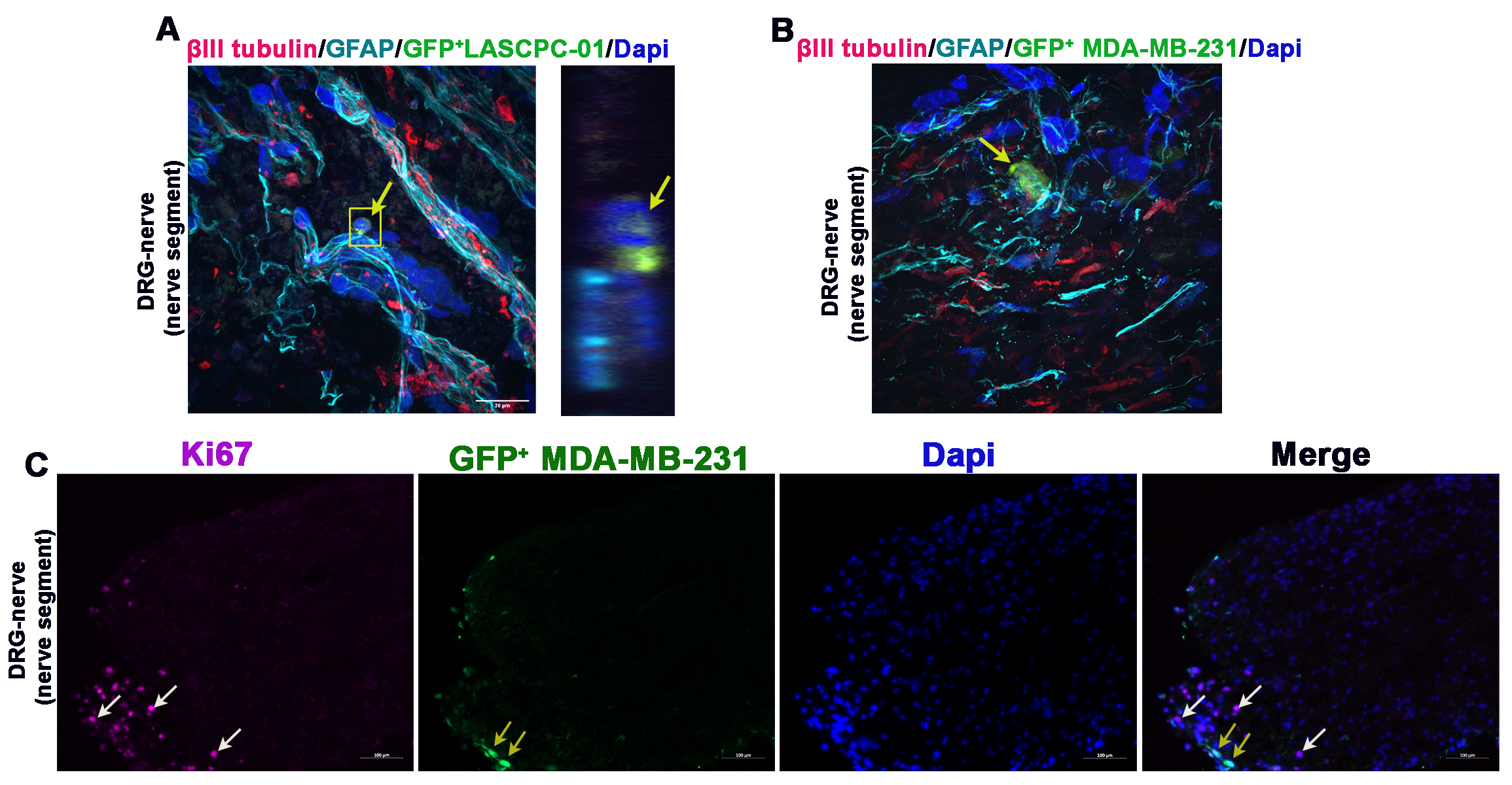

Supplement: Supplementary file 3 — Figure S2 [file 41420_2025_2616_MOESM3_ESM.tif]

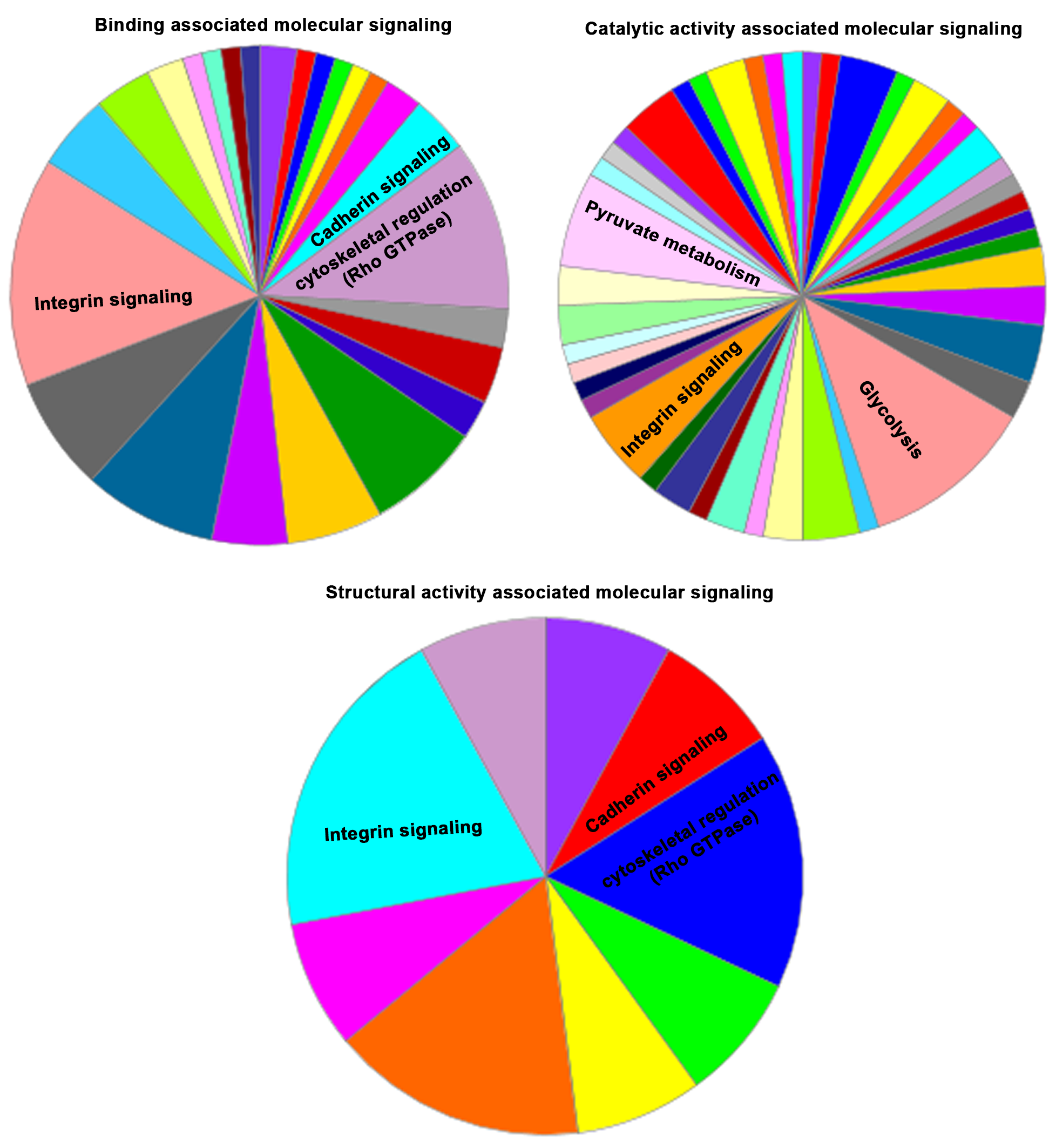

Supplement: Supplementary file 4 — Figure S3 [file 41420_2025_2616_MOESM4_ESM.tif]

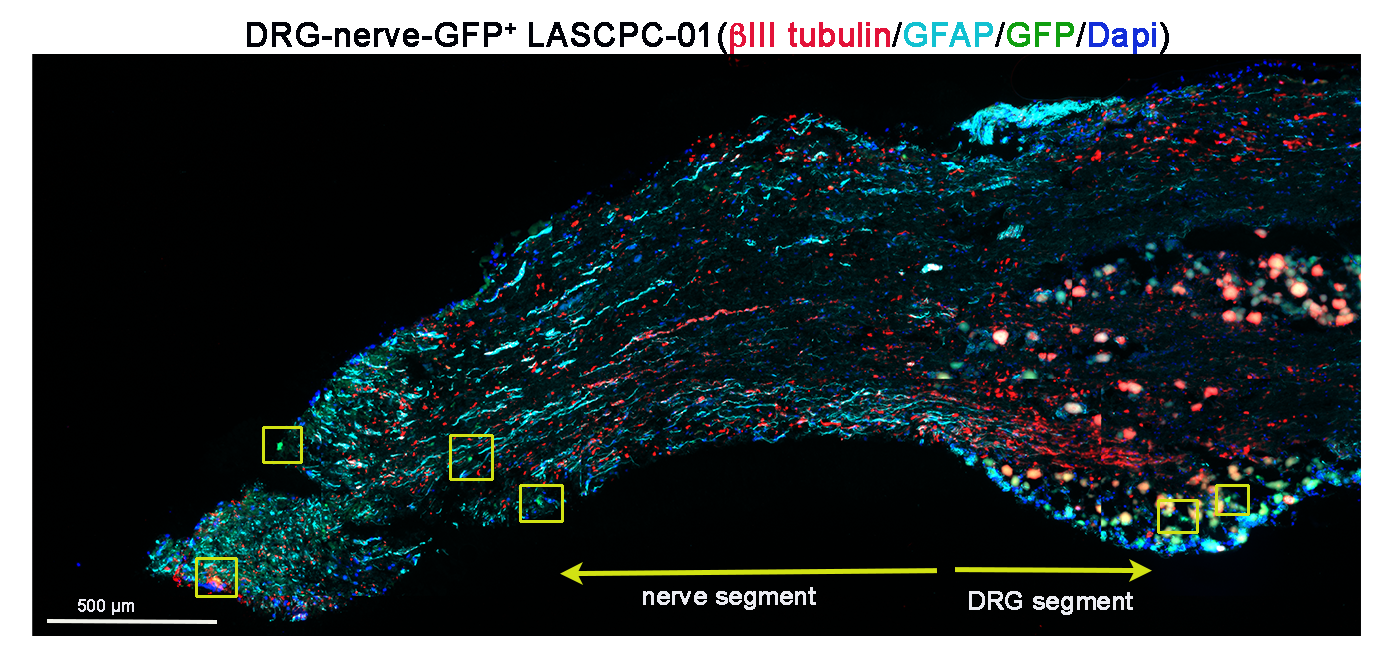

Supplement: Supplementary file 5 — Figure S4 [file 41420_2025_2616_MOESM5_ESM.tif]
