# Supplementary material for: Defining the cellular and molecular features of nerve-invaded cancer cells using a newly characterized experimental model
Source: Cell Death Discov. 2025 Jul 8;11:314. doi: 10.1038/s41420-025-02616-4 (PMC12238365; doi:10.1038/s41420-025-02616-4)
Supplement: Supplementary file 6 — Supplemental information [file 41420_2025_2616_MOESM6_ESM.docx]

**Supplemental information**

**Figure S1:** (**A**) Representative images of a section of the DRG-nerve preparation isolated from the PNI model (2-week time point) show the distribution of GFP^+^ prostate cancer cells (GFP^+^ LASCPC-01; white arrows) within the nerve segment (scale bar, 50µm). Schwann Cells (SCs) are marked with GFAP staining. The enlarged inset of the merged image shows a representative field within the nerve where a PNI cell (white arrow) interacts with SCs (yellow arrow). (**B**) Immunostaining shows the distribution of NF200^+^ and βIII tubulin^+^ axons and GFAP^+^ SCs in a normal sciatic nerve (scale bar, 100µm).

**Figure S2:** (**A**) A representative confocal image of an immunostained section of a DRG-nerve preparation from 14-day PNI model shows the co-localization of a PNI cell (GFP^+^ LASCPC-01) and Dapi (arrow); (left) A full view of the field is shown (scale bar, 20µm); (right) 3-Dimensional tilted and enlarged view of the boxed region of the stacked image shown on the left. (**B**) A representative confocal image of an immunostained section of a DRG-nerve preparation from 14-day PNI model shows the co-localization of a PNI cell (GFP^+^ MDA-MB-231) and Dapi (arrow) (the related stack of images showing the co-localization of GFP^+^ MDA-MB-231 and Dapi is shown in Movie S1). (**C**) Representative images of an immunostained section of a DRG-nerve preparation from 14-day PNI model shows no co-localization of Ki67 (cell proliferation marker; white arrows) and PNI cells (yellow arrows).

**Figure S3:** Pie charts show that signaling cascades associated with integrin and cadherin signaling and cytoskeletal regulation, and molecules associated with pyruvate and glucose metabolism are downregulated in PNI cells.

**Figure S4:** A full length view of an immunostained section of a DRG-nerve preparation from 14-day old PNI model. The DRG and nerve segments are indicated. The boxed regions show the presence of PNI cells (GFP^+^ LASCPC-01) in the nerve and DRG segments. Scale bar, 500µm.

**Movie S1:** The ‘Z’ stack images of an immunostained section of a DRG-nerve preparation from 14-day PNI model (shown in Figure S2B) shows the co-localization of GFP^+^ MDA-MB-231 and Dapi.

**Table S1:** Excel file shows the list of differentially regulated proteins in PNI cells identified from the proteomics experiments.

**Table S2:** Excel file shows the clinical parameters of the human breast cancer samples used for the study.
